# De novo entecavir+adefovir dipivoxil+lamivudine triple-resistance mutations resulting from sequential therapy with adefovir dipivoxil, and lamivudine

**DOI:** 10.1186/s12941-016-0138-0

**Published:** 2016-04-14

**Authors:** Song Yang, Huichun Xing, Qi Wang, Xiaomei Wang, Shunai Liu, Jun Cheng

**Affiliations:** Center of Hepatology, Beijing Ditan Hospital, Capital Medical University, 8 East Jingshun Street, Chaoyang District, Beijing, 100015 China; Institute of Infectious Diseases, Beijing Ditan Hospital, Capital Medical University, 8 East Jingshun Street, Chaoyang District, Beijing, 100015 China

**Keywords:** Hepatitis B virus, Multidrug resistance, Entecavir, Adefovir dipivoxil, Lamivudine

## Abstract

**Background:**

Entecavir-resistance mutations are commonly induced by entecavir treatment in chronic hepatitis B patients. However, entecavir+adefovir dipivoxil+lamivudine triple-resistance mutations induced by sequential or combination treatment with lamivudine and adefovir dipivoxil have never been reported.

**Results:**

We retrospectively reviewed 1200 patients who had been tested for anti-HBV drug resistance at Beijing Ditan Hospital of Capital Medical University, and five patients showing multidrug resistance to lamivudine and adefovir dipivoxil were enrolled. Stored serum samples were used for genetic analysis, which yielded a total of 135 clones. Entecavir+adefovir dipivoxil+lamivudine triple-resistance mutations were identified in 60 % (3/5) entecavir-naïve patients who received sequential therapy with adefovir dipivoxil and lamivudine. Specifically, we found one rtM204I+rtL180 M+rtM250 V+rtA181 V clone among 23 clones from patient 1 (4.35 %), one rtM204 V+vrtL180 M +rtM250 V+rtA181 V clone among 24 clones from patient 2 (4.17 %), and 2 clones harboring rtM204 V+rtL180 M+rtM250 V+rtA181 V and rtM204 V+rtL180 M+rtI169 V+rtA181 V among 20 clones from patient 3 (10.0 %). The other 2 patients showed multidrug resistance after lamivudine/telbivudine and adefovir dipivoxil combination therapy, but no entecavir-resistance mutations were found in these two patients.

**Conclusion:**

De novo entecavir+adefovir dipivoxil+lamivudine triple-resistance mutations can be induced by sequential therapy with adefovir dipivoxil and lamivudine in patients who never take entecavir. These results provide important information for sequential therapy with adefovir dipivoxil and lamivudine and the use of entecavir as a rescue therapy for these patients with multidrug resistance.

**Electronic supplementary material:**

The online version of this article (doi:10.1186/s12941-016-0138-0) contains supplementary material, which is available to authorized users.

## Findings

Chronic hepatitis B virus (HBV) infection is estimated to affect approximately 120 million people in China, with an annual death toll of approximately 300,000, mostly resulting from HBV-related cirrhosis and hepatocellular carcinoma (HCC) [1]. Although antiviral therapy with nucleoside/nucleotide analogues (NAs) and interferons (IFNs) can be used to suppress HBV replication and prevent disease progression, the side effects of antiviral drugs should be monitored regularly [2, 3]. Five NAs are currently approved for anti-HBV treatment in China, including lamivudine (LAM), adefovir dipivoxil (ADV), telbivudine (LdT), entecavir (ETV), and tenofovir disoproxil fumarate (TDF). One major concern for NA therapies is the emergence of HBV resistance after prolonged treatment, especially for drugs with low resistance barriers (LAM, ADV, and LdT) [2, 3]. In some patients, mutations causing multidrug resistance (MDR) may occur after sequential monotherapy with low-resistance barrier NAs [3]. Yim et al. reported three cases of LAM+ADV-resistant patients after sequential therapy with LAM and ADV [4]. Liu et al. detected a ETV+ADV+LAM triple-resistant HBV strain in a LAM+ADV-resistant patient who had taken sequential ETV as a rescue therapy [5]. Additionally, Kim et al. reported six cases of ETV+ADV+LAM resistance that occurred after sequential therapy with LAM, ADV, and ETV [6].

ETV resistance mutations are commonly induced by ETV treatment. However, Yang et al. reported that LAM-resistant HBV also had reduced susceptibility to ETV (37- to 471-fold reduction) [7]. Additionally, Inoue et al. described a patient who received LAM+ADV and then developed de novo ETV resistance with rtM204 V+rtL180 M+rtT184 S mutations [8]. However, it is still not clear whether or not sequential or combination therapy with LAM and ADV induces ETV+ADV+LAM triple-resistance mutations. Furthermore, ETV+ADV is still used for rescue therapy in many LAM+ADV-resistant patients [9–11], especially in China where TDF is costly and not covered by the reimbursement system. Therefore, we retrospectively analyzed chronic hepatitis B (CHB) patients in Beijing Ditan Hospital of Capital Medical University in whom sequential or combination therapy with LAM+ADV was not successful. Genetic analyses of stored serum samples revealed ETV+ADV+LAM triple-resistance mutations in three ETV-naïve patients who received sequential therapy with ADV+LAM.

The initial study population consisted of 1200 CHB patients who underwent drug resistance testing by nested polymerase chain reaction (PCR)-based direct sequencing at the Beijing Ditan Hospital. Written consent was obtained from each patient. Demographic and clinical data were collected using a questionnaire. Serum samples were stored at −20 °C until further analysis. Patients exhibiting MDR to both LAM and ADV were selected for the study. Clinical data were confirmed by checking medical records in the hospital information system. This study was approved by ethics committee of Beijing Ditan Hospital.

HBV DNA extraction was conducted using an AxyPrep Body Fluid Viral DNA/RNA Miniprep Kit (Corning Inc., Corning, NY). Nested PCR-direct sequencing was performed as previously described [12]. The PCR products were purified using a QIAquick PCR purification Kit (Qiagen, Valencia, CA, USA) according to the manufacturer’s instructions. Purified DNA was sequenced using an automated ABI 3730 DNA sequencer (Applied Biosystems, Foster City, CA, USA) at Beijing Augct Bioengineering Co., Ltd. DNA sequences were aligned using SeqMan and EditSeq software (DNASTAR Inc., Madison, WI). The HBV polymerase sequencing results were also used for HBV genotyping with the genotyping tool of the website of the National Center for Biotechnology Information [13]. PCR-amplified HBV DNA was cloned into the pGEM-T easy vector (Promega, Madison, WI) according to the manufacturer’s instructions. Clones (n = 20–38) were selected from each patient, and the sequences were analyzed using MegAlign software (DNASTAR Inc.).

Five HBV patients with MDR to both LAM/LdT and ADV were enrolled. The demographic and clinical characteristics of the patients, together with the direct sequencing results of the nested PCR products, are shown in Table 1. HBV genotyping showed that all 5 patients were infected with genotype C HBV.

**Table 1:** Demographic and clinical characteristics of patients

Patient	1	2	3	4	5
Age (years)	61	36	55	38	40
Sex	F	M	M	M	M
Race	Asian	Asian	Asian	Asian	Asian
Diagnosis	CHB	CHB	HBV-related cirrhosis, HCC	CHB	CHB
HBV genotype	C	C	C	C	C
HBeAg/Anti-HBe	−/+	+/−	+/−	+/−	+/−
Antiviral treatment history (months)	ADV (36) ↓^a^ LAM (12)	ADV (36) ↓ LAM (12)	ADV (12) ↓ LAM (12)	ADV (12) ↓ LAM+ADV (8)	LAM (36) ↓ LAM+ADV (8) ↓ LdT (6)
Results of sequencing	rtM204 V+rtL180 M+rtA181 V	rtM204 V+rtL180 M+rtA181 V	rtM204 V+rtL180 M+rtA181 V	rtM204 I+rtA181 V/T	rtM204 V+rtL180 M+rtA181 V

*ADV* adefovir dipivoxil, *LAM* lamivudine, *LdT* telbivudine, *PCR* polymerase chain reaction, *CHB* chronic hepatitis B, *HCC* hepatocellular carcinoma, *LAM* + *ADV* LAM and ADV combination therapy

^a^↓ indicates followed by

**Fig. 1:**
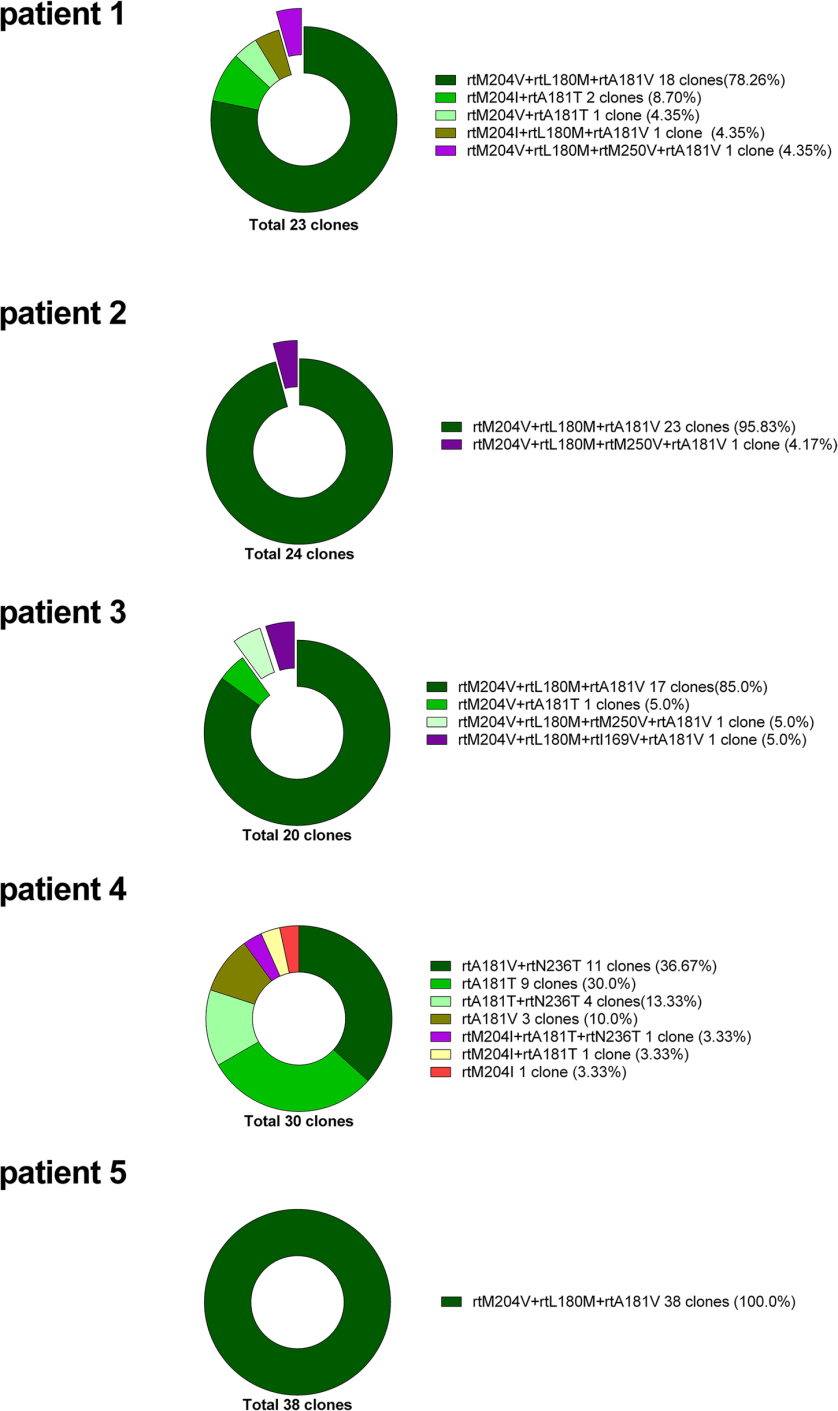
Genetic analysis of multidrug-resistant strains (135 clones) from patients receiving nucleoside/nucleotide analogue treatment. The expanded sections show de novo entecavir resistance mutations

In total, 135 clones were obtained from the five patients. All clones were submitted to NCBI GenBank, and the accession numbers are shown in Additional file 1: Table S1. The results of the clonal analysis are shown in Fig. 1. Patient 1 received ADV and LAM sequential therapy, and direct sequencing of the nested PCR product showed rtM204 V+rtL180 M+rtA181 V mutations upon secondary virological breakthrough, whereas rtM204 V+rtL180 M+rtA181 V strains accounted for 78.26 % (18/23) of the clones in the clonal analysis. In addition, 17.39 % (4/23) and 13.04 % (3/23) of the clones harbored the rtM204 I and rtA181 T mutations, respectively, which were not found by direct sequencing. Moreover, one clone (4.35 %) harbored rtM204 I+rtL180 M+rtM250 V+rtA181 V mutations, which resulted in resistance to LAM+ADV+ETV (Additional file 2: Figure S1); however, this patient had never received ETV therapy.

Patient 2 received ADV and LAM sequential therapy, and sequencing revealed rtM204 V+rtL180 M+rtA181 V mutations upon secondary virological breakthrough, whereas rtM204 V+rtL180 M+rtA181 V strains comprised 95.83 % (23/24) of the clones in the clonal analysis. Additionally, one clone (4.17 %) bore rtM204 V+rtL180 M+rtM250 V+rtA181 V mutations, which resulted in resistance to LAM+ADV+ETV (Additional file 3: Figure S2). This patient also never received ETV therapy.

Patient 3 received ADV and LAM sequential therapy, and sequencing showed rtM204 V+rtL180 M+rtA181 V mutations upon secondary virological breakthrough, whereas 85.0 % (17/20) of the clones harbored rtM204 V+rtL180 M+rtA181 V mutations. In addition, one clone (5.0 %) bore rtM204 V+rtA181 T mutations. Moreover, two clones (10.0 %) bore ETV+LAM+ADV triple-resistance mutations (Additional file 4: Figure S3 and Additional file 5: Figure S4), even though this patient had also never received ETV therapy.

Patient 4 received ADV+LAM for rescue therapy upon ADV resistance, and sequencing showed rtM204 I+rtA181 V+rtA181 T mutations upon secondary virological breakthrough. A high diversity of mutated strains was observed in the clonal analysis, with seven mutation patterns present in 30 clones. The rtN236 T mutation was not found by direct sequencing of PCR products, and no ETV-resistance mutations were detected in this patient.

Patient 5 received ADV+LAM for rescue therapy upon LAM resistance, but was later switched to LdT monotherapy, which was requested by the patient because of concerns regarding creatinine elevation. Sequencing identified rtM204 V+rtL180 M+rtA181 V mutations upon secondary virological breakthrough. All clones harbored rtM204 V+rtL180 M+rtA181 V mutations, consistent with the sequencing results, and no de novo ETV resistance mutations were detected.

The possible overlapping S-gene mutations of all clones harboring the rtA181 T mutation were analyzed (Additional file 6: Table S2). In patient 1, 3 clones harboring the rtA181 T mutation had sW172*(stop codon) mutations in the overlapping S gene. Additionally, 1 clone and 15 clones harboring rtA181 T mutations in in patients 3 and 4, respectively, showed sW172* mutations.

In this study, we performed a genetic analysis with serum samples obtained from five patients with chronic HBV infection that exhibited MDR to LAM/LdT and ADV following sequential/combination therapy with LAM/LdT and ADV. Surprisingly, de novo ETV resistance mutations were present in all three patients who received sequential therapy with ADV+LAM. Inoue et al. previously performed a clonal analysis and reported that strains with ETV resistance harbored only ETV resistance mutations and no ADV resistance mutations. However, in the present study, all three patients had HBV strains with MDR for ETV+LAM+ADV, although these MDR clones comprised only a minor population of the quasispecies.

There are several reports of the use of ETV as a rescue therapy for patients with MDR to LAM+ADV. However, Heo et al. reported that ETV monotherapy is inferior as a rescue therapy in patients with MDR to LAM and ADV [14]. Additionally, Xu et al. reported the use of ETV+ADV for rescue therapy in 45 patients who failed to respond to treatment with multiple NAs, with 2/45 patients showing LAM+ADV resistance at baseline. However, after 24 months of treatment with ETV+ADV treatment, one patient still did not achieve a complete virological response (HBV DNA ≤500 copies/mL) (11). Moreover, Lim et al. reported that ETV + ADV could be used as a rescue therapy in patients with a suboptimal response to LAM+ADV; however, only 28.9 % (13/45) of the patients achieved virological responses (HBV DNA < 60 IU/mL) after 52 months of therapy [15]. Notably, this study also included two patients with de novo ETV resistance mutations at rtT184 A and rtM250 L after LAM+ADV treatment, but no further clonal analyses were conducted in these patients. According to these studies, de novo ETV-resistance mutations after sequential and/or combination therapy with LAM+ADV may play a role in the inferior efficacy of ETV rescue therapy in these patients.

The present study also indicates a need for more sensitive HBV drug resistance tests in clinical practice. Direct sequencing of PCR products is frequently used in clinical practice and trials to detect NA resistance [16, 17]; however, it can only identify mutations when they reach approximately 20 % of the total HBV quasispecies pool [18]. As shown in this study, direct sequencing of PCR products was unable to detect de novo ETV-resistant strains in patients 1, 2, and 3, as these strains comprised 4.35, 4.17, and 15.0 % of the total quasispecies, respectively. However, minor mutant strains can evolve to major strains under ETV selection pressure, and thus their early detection is important. More sensitive resistance tests have been reported to detect mutant strains comprising <5 % of the total HBV quasispecies, but they are either costly or inconvenient for widespread use in China [19, 20].

The study also calls attention to the use of less potent NAs in combination as a rescue therapy for NA resistance [2, 3]. Addition of LAM is a therapeutic option for ADV-resistant patients. In this study, patient 4 developed MDR to LAM+ADV while receiving LAM+ADV combination therapy to rescue ADV resistance. Other reports have described similar phenomena [8, 15]. Thus, thorough and rapid inhibition of HBV replication is critical for combination therapy; otherwise, combination therapy with LAM+ADV may increase the selection pressure of both drugs in patients with persistent viremia.

In this study, all MDR patients were infected with genotype C. we consider this is partly because genotype C is predominant in the northern part of China [1]. Several reports have indicated higher numbers of ADV or LAM resistance mutations in HBV genotype C compared with genotype B [21, 22]. However, the sample size was too small to establish a correlation between genotype C and MDR mutations.

It has been reported that the rtA181 T mutation in HBV may cause sW172* in the overlapping S gene in genotype D HBV infection [23]. In this study, all genotype C HBV clones harboring the rtA181 T mutation had sW172* mutations in the overlapping S-gene. Strains harboring the rtA181 T/sW172* mutation accounted for 13.04 % (3/23), 4.17 % (1/24), and 50.0 % (15/30) of the total clones in patients 1, 3, and 4, respectively. This also confirms that the HBV rtA181 T/sW172*mutation is usually detected in a mixed population with lower replication efficiency [24].

In conclusion, our results indicate that de novo LAM+ADV+ETV resistance mutations may be induced by sequential therapy with ADV+LAM in patients who never took ETV. These results provide important information for administration of sequential therapy with ADV and LAM and for the use of ETV for rescue therapy in patients with MDR to ADV+LAM.

## Additional files

10.1186/s12941-016-0138-0 GenBank accession numbers of 135 sequences from 5 chronic hepatitis B patients.
10.1186/s12941-016-0138-0 Electropherogram of rtM204 V+rtL180 M+rtA181 V+rtM250 V clone in Patient 1 (GenBank accession number: KU736795).
10.1186/s12941-016-0138-0 Electropherogram of rtM204 V+rtL180 M+rtA181 V+rtM250 V clone in Patient 2 (GenBank accession number: KU751680).
10.1186/s12941-016-0138-0 Electropherogram of rtM204 V+rtL180 M+rtA181 V+rtI169 V clone in Patient 3 (GenBank accession number: KU751729).
10.1186/s12941-016-0138-0 Electropherogram of rtM204 V+rtL180 M+rtA181 V+rtM250 V clone in Patient 3 (GenBank accession number: KU751733).
10.1186/s12941-016-0138-0 Overlapping HBsAg mutations caused by HBV rtA181 T mutation.

## Abbreviations

HBV: hepatitis B virus
MDR: multidrug resistance
LAM: lamivudine
ADV: adefovir dipivoxil
LdT: telbivudine
ETV: entecavir
TDF: tenofovir disoproxil fumarate
NA: nucleoside/nucleotide analogue
