# Supplementary material for: De novo entecavir+adefovir dipivoxil+lamivudine triple-resistance mutations resulting from sequential therapy with adefovir dipivoxil, and lamivudine
Source: Ann Clin Microbiol Antimicrob. 2016 Apr 14;15:24. doi: 10.1186/s12941-016-0138-0 (PMC4832522; doi:10.1186/s12941-016-0138-0)
Supplement: Supplementary file 1 — 10.1186/s12941-016-0138-0 GenBank accession numbers of 135 sequences from 5 chronic hepatitis B patients. [file 12941_2016_138_MOESM1_ESM.doc]

Tabel S1. GenBank accession numbers of 135 sequences from 5 chronic hepatitis B patients.

| **Patient number** | **NA resistance mutation type (N)** | **GenBank accession number** |
| --- | --- | --- |
| 1 | rtM204V+rtL180M+rtM250V+rtA181V(1) | KU736795 |
|  | rtM204I+rtL180M+rtA181V(1) | KU751765 |
|  | rtM204V+rtA181T(1) | KU751763 |
|  | rtM204I+rtA181T(2) | KU751755, KU751746 |
|  | rtM204V+rtL180M+rtA181V(18) | KU728064, KU751747, KU751748, KU751749, KU751750, KU751751, KU751752, KU751753, KU751754, KU751756, KU751757, KU751758, KU751759, KU751760, KU751761, KU751762, KU751764, KU751766 |
| 2 | rtM204V+rtL180M+rtM250V+rtA181V(1) | KU751680 |
|  | rtM204V+rtL180M+rtA181V(23) | KU751672, KU751673, KU751674, KU751675, KU751676, KU751677, KU751678, KU751679, KU751681, KU751682, KU751683, KU751684, KU751685, KU751686, KU751687, KU751688, KU751689, KU751690, KU751691, KU751692, KU751693, KU751694, KU751695 |
| 3 | rtM204V+rtL180M+rtM250V+rtA181V(1) | KU751733 |
|  | rtM204V+rtL180M+rtI169V+rtA181V(1) | KU751729 |
|  | rtM204V+rtA181T(1) | KU751738 |
|  | rtM204V+rtL180M+rtA181V(17) | KU751726, KU751727, KU751728, KU751730, KU751731, KU751732, KU751734, KU751735, KU751736, KU751737, KU751739, KU751740, KU751741, KU751742, KU751743, KU751744, KU751745 |
| 4 | rtM204I+rtA181T+rtN236T(1) | KU751697 |
|  | rtM204I(1) | KU751699 |
|  | rtA181V(3) | KU751708, KU751709, KU751704 |
|  | rtM204I+rtA181T(1) | KU751720 |
|  | rtA181T+rtN236T(4) | KU751723, KU751725, KU751722, KU751696 |
|  | rtA181T(9) | KU751700, KU751703, KU751705, KU751710, KU751713, KU751715, KU751716, KU751721, KU751714 |
|  | rtA181V+rtN236T(11) | KU751698, KU751702, KU751706, KU751707, KU751711, KU751712, KU751717, KU751718, KU751719, KU751724, KU751701 |
| 5 | rtM204V+rtL180M+rtA181V(38) | KU746290, KU746291, KU746292, KU746293, KU746294, KU746295, KU746296, KU746297, KU746298, KU746299, KU746300, KU746301, KU746302, KU746303, KU746304, KU746305, KU746306, KU746307, KU746308, KU746309, KU746310, KU746311, KU746312, KU746313, KU746314, KU746315, KU746316, KU746317, KU746318, KU746319, KU746320, KU746321, KU746322, KU746323, KU746324, KU746325, KU746326, KU746327 |

Abbreviations: NAs, nucleoside/tide analogues.
