# Supplementary figures and images for: De novo entecavir+adefovir dipivoxil+lamivudine triple-resistance mutations resulting from sequential therapy with adefovir dipivoxil, and lamivudine
Source: Ann Clin Microbiol Antimicrob. 2016 Apr 14;15:24. doi: 10.1186/s12941-016-0138-0 (PMC4832522; doi:10.1186/s12941-016-0138-0)

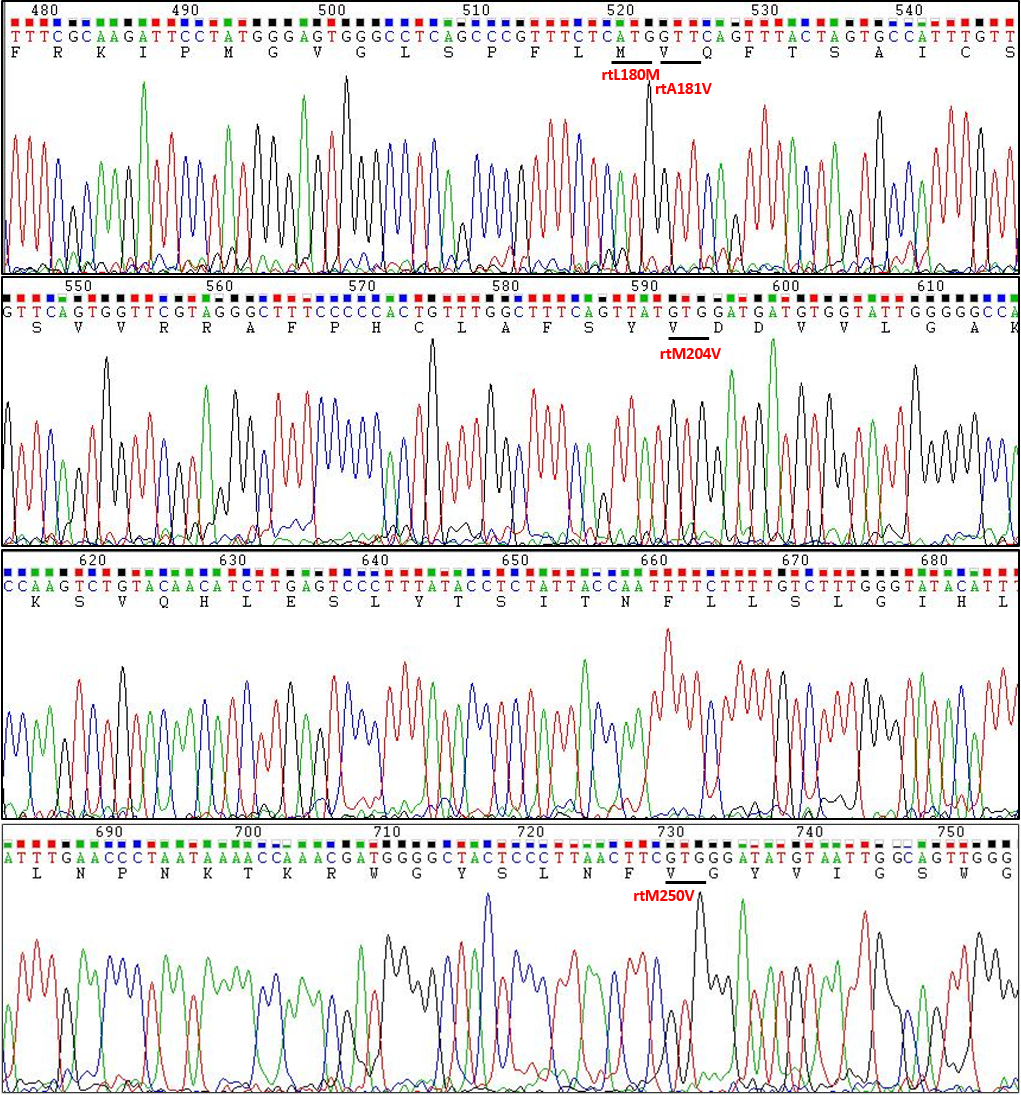

Supplement: Supplementary file 2 — 10.1186/s12941-016-0138-0 Electropherogram of rtM204 V+rtL180 M+rtA181 V+rtM250 V clone in Patient 1 (GenBank accession number: KU736795). [file 12941_2016_138_MOESM2_ESM.tif]

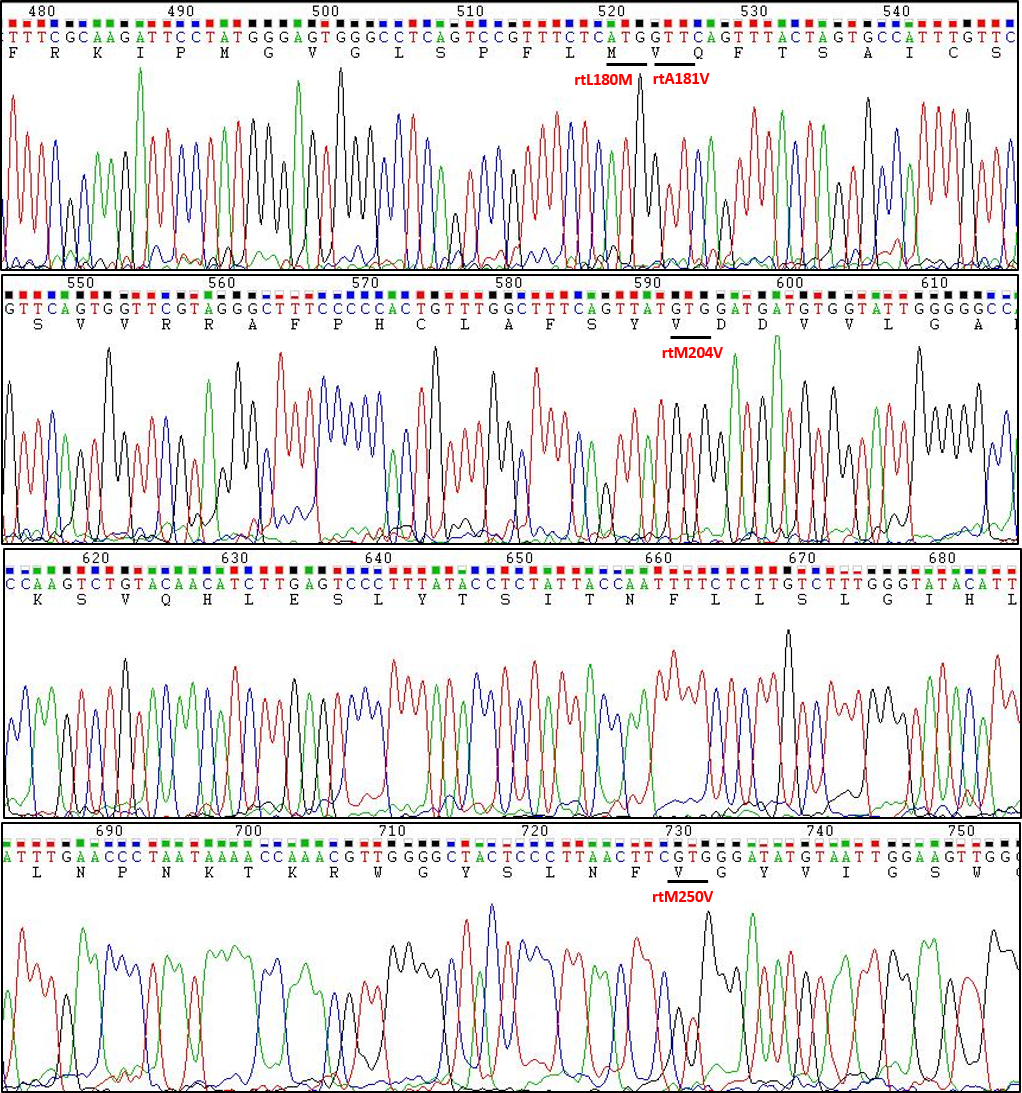

Supplement: Supplementary file 3 — 10.1186/s12941-016-0138-0 Electropherogram of rtM204 V+rtL180 M+rtA181 V+rtM250 V clone in Patient 2 (GenBank accession number: KU751680). [file 12941_2016_138_MOESM3_ESM.tif]

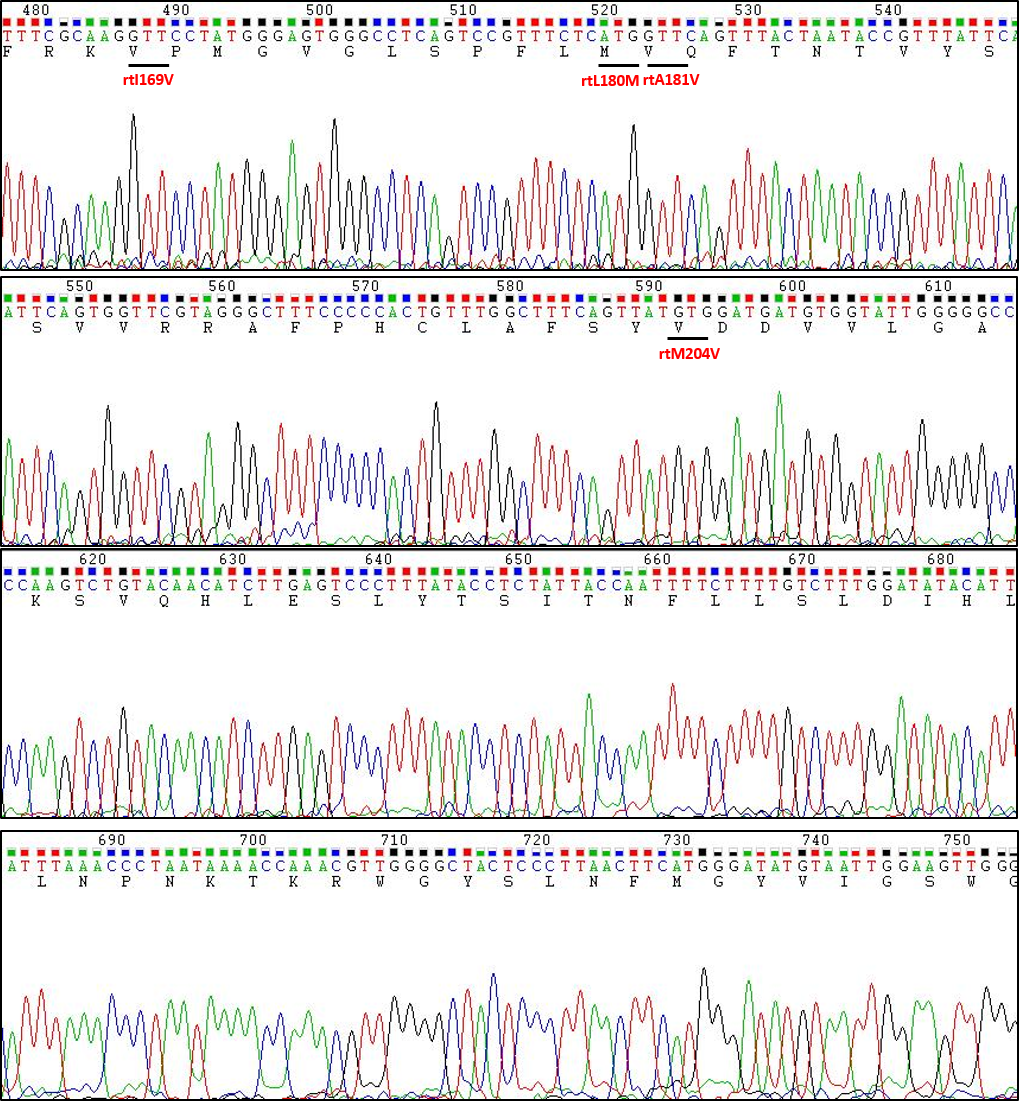

Supplement: Supplementary file 4 — 10.1186/s12941-016-0138-0 Electropherogram of rtM204 V+rtL180 M+rtA181 V+rtI169 V clone in Patient 3 (GenBank accession number: KU751729). [file 12941_2016_138_MOESM4_ESM.tif]

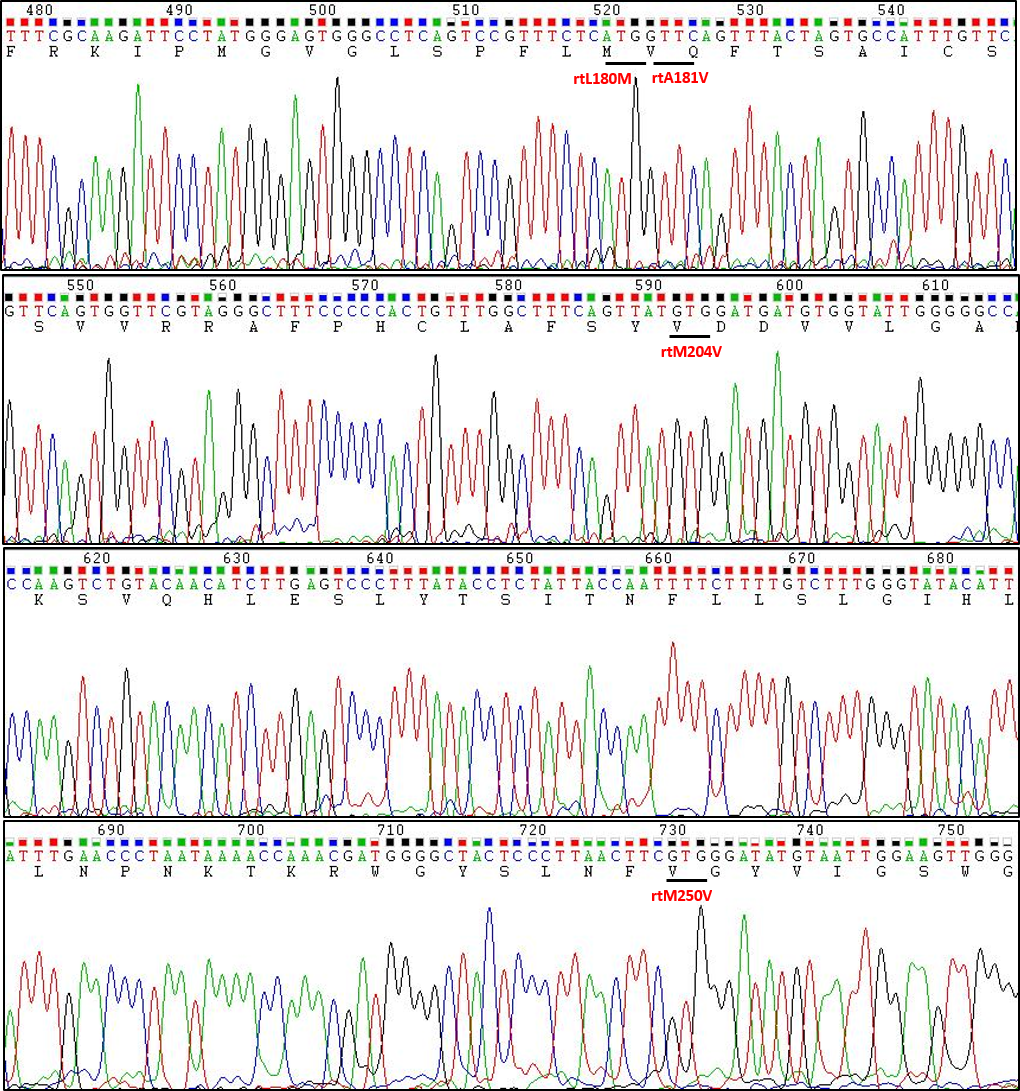

Supplement: Supplementary file 5 — 10.1186/s12941-016-0138-0 Electropherogram of rtM204 V+rtL180 M+rtA181 V+rtM250 V clone in Patient 3 (GenBank accession number: KU751733). [file 12941_2016_138_MOESM5_ESM.tif]
