# Supplementary material for: De novo entecavir+adefovir dipivoxil+lamivudine triple-resistance mutations resulting from sequential therapy with adefovir dipivoxil, and lamivudine
Source: Ann Clin Microbiol Antimicrob. 2016 Apr 14;15:24. doi: 10.1186/s12941-016-0138-0 (PMC4832522; doi:10.1186/s12941-016-0138-0)
Supplement: Supplementary file 6 — 10.1186/s12941-016-0138-0 Overlapping HBsAg mutations caused by HBV rtA181 T mutation. [file 12941_2016_138_MOESM6_ESM.doc]

Table S2. Overlapping HBsAg mutations caused by HBV rtA181T mutation.

| **Patient number** | **GenBank accession number of clones** | **HBV RT region mutations** | **Overlapping HBsAg mutations** |
| --- | --- | --- | --- |
| 1 | KU751763 | rtM204V+rtA181T | sW172*(stop codon) |
| 1 | KU751755 | rtM204I+rtA181T | sW172* |
| 1 | KU751746 | rtM204I+rtA181T | sW172* |
| 3 | KU751738 | rtM204V+rtA181T | sW172* |
| 4 | KU751697 | rtM204I+rtA181T+rtN236T | sW172* |
| 4 | KU751720 | rtM204I+rtA181T | sW172* |
| 4 | KU751725 | rtA181T+rtN236T | sW172* |
| 4 | KU751722 | rtA181T+rtN236T | sW172* |
| 4 | KU751696 | rtA181T+rtN236T | sW172* |
| 4 | KU751723 | rtA181T+rtN236T | sW172* |
| 4 | KU751700 | rtA181T | sW172* |
| 4 | KU751703 | rtA181T | sW172* |
| 4 | KU751705 | rtA181T | sW172* |
| 4 | KU751710 | rtA181T | sW172* |
| 4 | KU751713 | rtA181T | sW172* |
| 4 | KU751715 | rtA181T | sW172* |
| 4 | KU751716 | rtA181T | sW172* |
| 4 | KU751721 | rtA181T | sW172* |
| 4 | KU751714 | rtA181T | sW172* |

Abbreviations: NA, nucleoside/tide analogue; HBV, hepatitis B virus; HBsAg, hepatitis B virus surface antigen; RT, reverse transcriptase.
